# A monster of a disease

**DOI:** 10.7554/eLife.75862

**Published:** 2021-12-13

**Authors:** Sahana Sitaraman

**Affiliations:** 1 National Centre for Biological Sciences Bangalore India

**Keywords:** sparks of change, research culture, mental health

## Abstract

When compulsions and obsessive thoughts took over her world, a graduate student found strength in her identity as a scientist.

I’m bathed and dressed, sitting on my bed in my impeccably clean hostel room, ready to go to the lab. But my mind won’t let me.

I wrap my arms around myself, sweating, feeling the familiar ache in the pit of my stomach. The thought of stepping outside and taking the crowded bus to campus paralyses me with fear. I don’t care that my experiment samples will go to waste. I don’t care that without this data I’ll have nothing to present. I don’t even care that I won’t show up for the special dinner my lab mates have planned. I need to stay in my bubble, where nothing can harm me, no one can come near me. It doesn’t take much to convince myself to stay put, away from all my triggers. I spend the day cleaning my already spotless room, taking another bath, washing my hands every chance I get. They are raw and cracked by now.

I wasn’t always like this. I used to poke around in mud looking for insects, explore new places on a whim, make friends with strangers. Carrying this sense of wonder into my early adult years, I had just started a PhD at the National Centre for Biological Sciences in Bangalore, India. No one, least of all me, expected I would spend the next few years consumed with obsessive, anxious and irrational thoughts. I was yet to understand how being a scientist would keep me locked inside the disease, before finally offering me the key to recovery.

**Figure 1:**
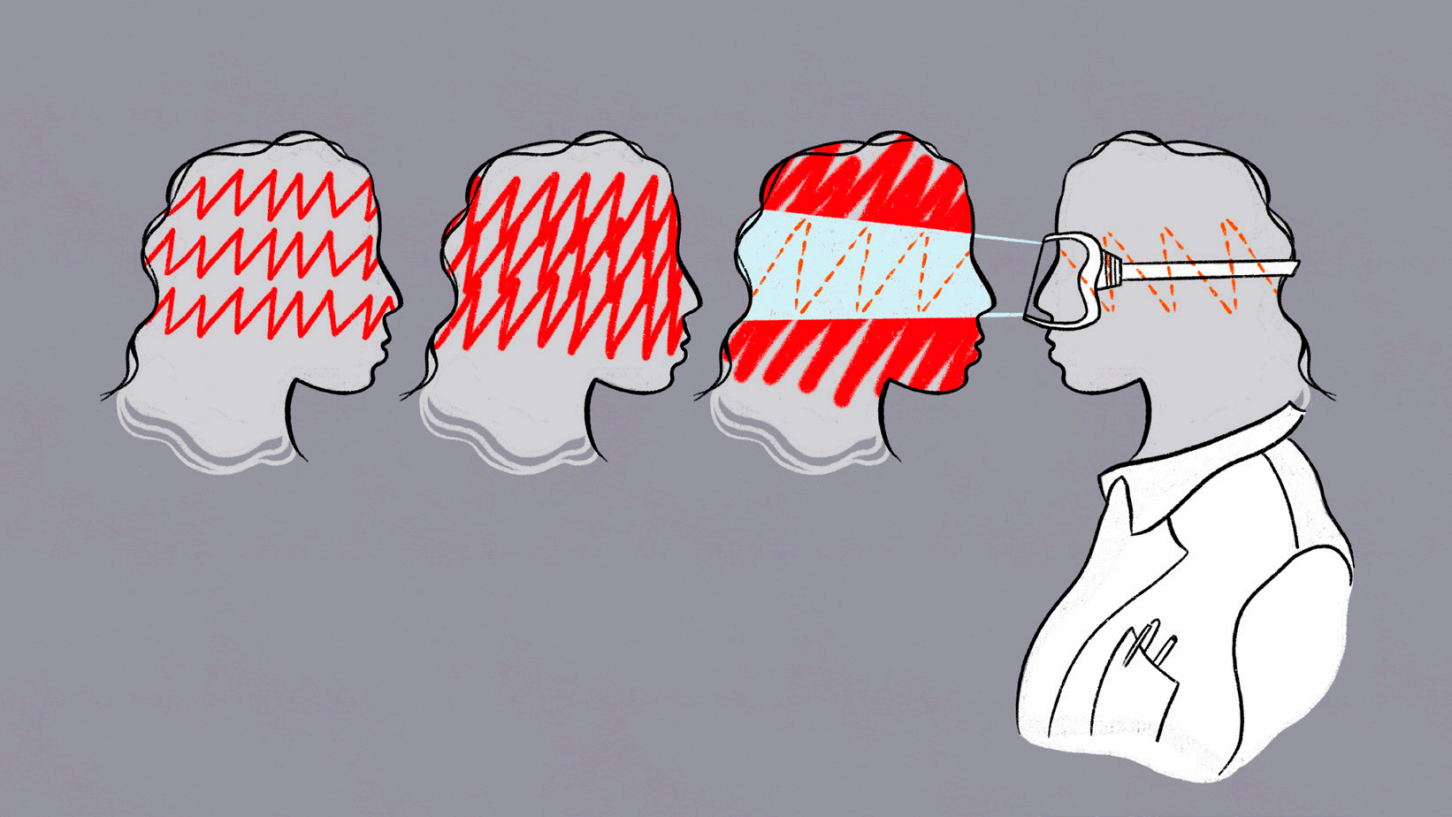
After becoming consumed by obsessive, anxious and irrational thoughts, a graduate student harnessed her scientific mindset to create a path towards a healthier future.

IMAGE CREDIT: Carys Boughton; http://www.carysboughton.com (CC BY 4.0).

It started out small; washing my hands for a few extra seconds, avoiding places that looked unclean, leaving my shoes by the door of my room. All things one might do in the name of hygiene – but I didn’t stop to question why I needed them to feel calm. Gradually, the compulsions started to overflow; I crossed the thin line between healthy and compulsive habits and left it far behind without even realizing. There was nothing rational about the increasingly convoluted routes I was taking to steer clear of my growing list of ‘dirty’ places. There was no scientific reason for me to wash my hands if someone touched my chair, to rinse off my face if I saw garbage, to shut my eyes tight while the bus drove past a landfill so I wouldn’t feel I needed a bath.

And yet I used my training, first as a microbiologist and then as a neurobiologist, to defend and even justify these habits. I was certain that my behaviours stemmed from forming legitimate associations between specific situations and the harm they could cause me. After all, I had read that animals learn by linking together various stimuli; the more often these happen together, the stronger the association becomes. I convinced myself that my ‘hygiene rituals’ were acceptable because they would keep me from falling sick, reasoning that I was healthy only because my behaviours allowed me to stay that way. Engaging in these compulsions reinforced this link and my belief that my habits had a logical, scientific foundation. I latched onto this fact to explain away my compulsions long after it stopped making sense.

The illness spawned a vicious cycle of doubt and fear that started to seep into all aspects of my life, including those that had no effect on my health. My thoughts were always in a storm: “Did I lock the room? Let me just go back and turn the knob. It’s not opening. That’s good, I can go now. Wait, maybe the door opened when I turned the knob. Let me go and check again.” And again. And again. Doubting my instincts made me anxious, and I engaged in repetitive compulsions to relieve the anxiety: this sometimes provided momentary comfort, which only strengthened the pattern. At its peak, over half of my waking hours were spent scanning my surroundings for triggers and feeding this parasitic illness with the compulsions and mind-numbing habits it was craving. I was exhausted.

The turning point came when this monster of a disease robbed me of my ability to think, conduct research, and more importantly enjoy science. I draw a lot of pride from being a good scientist; I’m confident in my methods and results. But one morning, I sat down at my desk to analyse the data from a crucial experiment, and something was off. The results were fascinating, yet I was sure I had done something wrong. I spent the next week running the same code. Over, and over, and over again. I couldn’t trust the results because I had lost faith in myself, and I realized I had reached rock bottom.

I accepted that I had a mental disorder, but wasn’t ready to push it out of my life. Asking for help meant opening up and putting a stop to my compulsions. I didn’t have the strength yet to make the necessary changes; all I wanted was a magic pill that would make me feel better overnight. I lashed out at my partner when he provided me the push I desperately needed, only to feel guilty afterwards. Nevertheless he persisted, urged me to face my triggers, reassured me, and kept a keen eye on my progress. I improved, but slid back down as quickly. Going through this futile cycle finally made me decide to get professional help. The counsellor confirmed what I already knew subconsciously: I was exhibiting classic signs of obsessive compulsive disorder. She started to design a recovery plan that would work best for me.

Ultimately, however, my recovery really started when I stopped using science to justify my behaviour, and harnessed my scientific mindset to find solutions. I scientifically dissected my disease; I dived into papers, interviews, first-hand accounts, anything I could find that would help me better understand what I was going through. I learnt about the varied ways it manifests and that there isn’t a one-size-fits-all treatment. Based on my knowledge, I tailored the help I was getting to my specific triggers. I designed different approaches, compared the results and then tweaked the method based on the data. This helped me reclaim the scientist in me, and finally my whole self.

Today, I still live with the illness, but it doesn’t control me. It took me over two years of constantly reminding myself that not engaging in my compulsions will not harm me, drumming it in until it became second nature. Every day, I have to remember to keep habits under control, to not replace one obsession with another in the name of ‘becoming healthier’. Every day, with the help of my family, friends and support network, I channel the scientist in me to observe myself, take note of behaviours that don’t make sense, design solutions to stop them and find the inner strength to keep going.

I am now finishing my PhD and shifting gears to become a science journalist. I started graduate school thinking that scientists discover and invent within the confines of a lab. Now I know that the arduous part is applying that training and knowledge to problem-solve, innovate and move ahead in your everyday life. Shifting my understanding of what it means to be a scientist has made me a better researcher, and a much healthier person.

## Share your experiences

This article is a Sparks of Change column, where people around the world share moments that illustrate how research culture is or should be changing. Have an interesting story to tell? See what we’re looking for and the best ways to get in touch here.

